# State-Wide Genomic and Epidemiological Analyses of Vancomycin-Resistant *Enterococcus faecium* in Tasmania’s Public Hospitals

**DOI:** 10.3389/fmicb.2019.02940

**Published:** 2020-01-15

**Authors:** Kelvin W. C. Leong, Ranmini Kalukottege, Louise A. Cooley, Tara L. Anderson, Anne Wells, Emma Langford, Ronan F. O’Toole

**Affiliations:** ^1^Department of Pharmacy and Biomedical Sciences, School of Molecular Sciences, College of Science, Health and Engineering, La Trobe University, Albury-Wodonga, VIC, Australia; ^2^Department of Microbiology, Launceston General Hospital, Launceston, TAS, Australia; ^3^Royal Hobart Hospital, Hobart, TAS, Australia; ^4^School of Medicine, University of Tasmania, Hobart, TAS, Australia; ^5^Tasmanian Infection Prevention and Control Unit, Department of Health and Human Services, Hobart, TAS, Australia; ^6^Department of Microbiology, Hobart Pathology, Hobart, TAS, Australia; ^7^Department of Clinical Microbiology, Trinity College Dublin, Dublin, Ireland

**Keywords:** *Enterococcus faecium*, whole genome sequencing, vancomycin, multi-locus sequence typing, single nucleotide polymorphism

## Abstract

From 2015 onwards, the number of vancomycin-resistant *Enterococcus faecium* (VREfm) isolates increased in Tasmania. Previously, we examined the transmission of VREfm at the Royal Hobart Hospital (RHH). In this study, we performed a state-wide analysis of VREfm from Tasmania’s four public acute hospitals. Whole-genome analysis was performed on 331 isolates collected from screening and clinical specimens of VREfm. *In silico* multi-locus sequence typing (MLST) was used to determine the relative abundance of broad sequence types (ST) across the state. Core genome MLST (cgMLST) was then applied to identify potential clades within the ST groupings followed by single-nucleotide polymorphic (SNP) analysis. This work revealed that differences in VREfm profiles are evident between the state’s two largest hospitals with the dominant *vanA* types being ST80 at the RHH and ST1421 at Launceston General Hospital (LGH). A higher number of VREfm cases were recorded at LGH (*n* = 54 clinical, *n* = 122 colonization) compared to the RHH (*n* = 14 clinical, *n* = 67 colonization) during the same time period, 2014–2016. Eleven of the clinical isolates from LGH were *vanA* and belonged to ST1421 (*n* = 8), ST1489 (*n* = 1), ST233 (*n* = 1), and ST80 (*n* = 1) whereas none of the clinical isolates from the RHH were *vanA*. For the recently described ST1421, cgMLST established the presence of individual clusters within this sequence type that were common to more than one hospital and that included isolates with a low amount of SNP variance (≤16 SNPs). A spatio-temporal analysis revealed that VREfm *vanA* ST1421 was first detected at the RHH in 2014 and an isolate belonging to the same cgMLST cluster was later collected at LGH in 2016. Inclusion of isolates from two smaller hospitals, the North West Regional Hospital (NRH) and the Mersey Community Hospital (MCH) found that ST1421 was present in both of these institutions in 2017. These findings illustrate the spread of a recently described sequence type of VREfm, ST1421, to multiple hospitals in an Australian state within a relatively short time span.

## Introduction

Vancomycin-resistant *Enterococcus faecium* (VREfm) is an important antibiotic-resistant microorganism that can cause healthcare-associated infections (HAI) in patients receiving care. It was first recorded in Australia at a Melbourne hospital in 1994 (Kamarulzaman et al., 1995). By 2015, Australia exhibited one of the highest rates of vancomycin resistance in *E. faecium* in the world at 48.7–56.8% of clinical isolates (Australian Commission on Safety and Quality in Health Care (ACSQHC), 2017). From 2008 to 2015, the prevalence of VREfm in Tasmania was relatively low with an average of approximately 10 new VREfm isolates per quarter during that period (Wilson et al., 2017). However, by 2016 there was a marked increase to over 100 VREfm isolates collected on average per quarter (Wilson et al., 2017). The reasons underlying the abrupt rise in VREfm in the state have not yet been established. Previously, we applied whole-genome sequencing to examine VREfm at the Royal Hobart Hospital (RHH) and identified the major sequence types as *vanB* ST796 and *vanA* ST80 as well as their probable direction of transmission at the hospital (Leong et al., 2018a).

In this work, we examined VREfm on a state-wide basis to improve our understanding of this pathogen across Tasmania. We determined the genotypes of VREfm isolates collected at Tasmania’s other public hospitals, the Launceston General Hospital (LGH), the North West Regional Hospital (NRH) and the Mersey Community Hospital (MCH), using multi-locus sequence typing (MLST), core genome MLST (cgMLST), and single-nucleotide polymorphic (SNP) analysis. We then combined genomic data with patient spatio-temporal information which provided insights into the emergence and distribution of VREfm sequence types in the state.

## Materials and Methods

### VREfm Isolate and Epidemiological Data Collection

The Multi-Resistant Organism Screening and Clearance Protocol for the Tasmanian Health Services identifies VRE colonization in patients when a VRE-positive culture was obtained from a non-sterile site and VRE-specific antibiotic therapy was not administered by a clinician, and identifies VRE infection when a VRE-positive culture was obtained from either a sterile or non-sterile site and VRE-specific antibiotic therapy was administered by a clinician (Wilson et al., 2018). In accordance with the Australian Public Health Act 1997 (Act Parliamentary Counsel, 2016), the Tasmanian Infection Prevention and Control Unit (TIPCU) of the Department of Health and Human Services (DHHS) established the Healthcare Associated Infection Surveillance Program for the notification of new patient cases with VRE (Wilson et al., 2018). VREfm screening isolates were obtained from inpatients who underwent VRE screening under the following circumstances: direct transfers from any intrastate, interstate or overseas acute or long-term healthcare facility; patients with an overnight admission in the previous 3 months to any intrastate acute or long term healthcare facility; patients with an overnight admission in the previous 12 months to any intrastate acute or overseas acute or long term healthcare facility; patients with a “History-VRE” alert; patients with a self-reported or healthcare facility reported history of VRE; or patients identified to be a VRE contact. When patients presented with an VRE infection, the VRE isolates were classified as clinical isolates (Wilson et al., 2018).

For whole-genome sequencing, we collected VREfm samples from Tasmania’s acute public hospitals based on the following criteria: all clinical isolates from 2014–2016, screening samples which overlapped with the clinical isolates collected with respect to patient admission and sample collection dates, and all VREfm that were tested positive for *vanA* vancomycin resistance. A total of 257 VREfm isolates including both clinical and screening cases, were retrieved from patient samples collected between 2014 and 2016 at the RHH (*n* = 500 beds approx.) and LGH (*n* = 300 beds approx.). Isolates from the state’s smaller hospitals, the NRH at Burnie (*n* = 160 beds approx.) and the MCH near Devonport (*n* = 95 beds approx.), were also included. Storage of VREfm isolates at these two hospitals did not commence until late 2016 and did not include clinical isolates during 2016. To investigate the epidemiology of the VREfm isolates from the NRH and MCH, a full calendar year of isolates (*n* = 74) collected in 2017 was analyzed.

Stored isolates were retrieved and cultured on blood agar plates at the Microbiology laboratories of the respective hospitals. Only one VREfm isolate per patient was included. At RHH and LGH, the Bruker Biotyper matrix assisted laser desorption ionization-time of flight mass spectrometry (MALDI-TOF MS) (Bruker Daltonic GmbH, Leipzig, Germany) was used to identify *E. faecium* and antibiotic susceptibility testing (AST) was performed using the EUCAST methodology^1^. For NRH and MCH, the bioMérieux Vitek MALDI-TOF MS (bioMérieux Australia Pty Ltd., Baulkham Hills, NSW, Australia) was used. AST was performed using the agar disc diffusion assay and zone diameters of inhibition were interpreted using the calibrated dichotomous sensitivity (CDS) test clinical breakpoint of 2 mm to differentiate between VRE sensitivity and resistance for *Enterococcus* species^2^. For all sites, identification of VRE was also determined by growth on VRE-selective agar, organism detection, and a final confirmation of the vancomycin-resistance locus type was obtained with the Cepheid Xpert^®^ *vanA*/*vanB* assay (Xpert^®^ *vanA*/*vanB*)^3^.

Information was collected from the hospital’s electronic medical record and infection control database which included patient admission and discharge dates, specimen type and collection date, patient ward location on date of specimen collection, and patient ward/hospital movements during hospitalization. Ethics approval for this study was obtained from the Tasmanian Health and Medical Human Research Ethics Committee (Reference# H0016214).

### Genomic DNA Purification

Enterococcal isolates were sub-cultured in thioglycolate broth (TM0935, 15 mL) (Thermo Fisher Scientific, Waltham, MA, United States) at LGH, RHH, and the Hobart Pathology Laboratory for NRH and MCH. The isolates were analyzed at the School of Medicine, University of Tasmania, and the School of Molecular Sciences, La Trobe University, Australia. The extraction and purification of genomic DNA was processed in accordance to the protocol previously described by Gautam et al. (2019). Briefly, 1.5 mL of broth culture was centrifuged, and the cell pellet was resuspended in a mixture of lysozyme [30 μL lysozyme (50 mg/mL)] (Muramidase, VWR Chemicals, Radnor, PA, United States) and phosphate buffered saline (PBS) (600 μL) and incubated at 37°C for 1 h. Using the DNeasy Blood and Tissue Kit protocol (Qiagen, Hilden, Germany), 200 μL of lysate was used to initially extract 100 μL of DNA eluate. This was treated with 2 μL of RNase (100 mg/mL) (Qiagen, Hilden, Germany), incubated at room temperature for 1 h, and further purified using the High Pure PCR Template Preparation Kit (Roche, Basel, Switzerland) to achieve a final 50 μL of DNA eluate. The Qubit 2.0 Fluorometer (Life Technologies, Carlsbad, CA, United States) was used with the Qubit dsDNA (double-stranded DNA) HS (high sensitivity) Quantification Kit to measure the DNA concentration before diluting with purified water to a concentration of 0.2 ng/μL (input DNA).

### DNA Library Preparation

The Nextera XT DNA Library Preparation Kit (Illumina Inc., San Diego, CA, United States) was used to generate the DNA libraries from an input DNA volume of 2.5 μL for whole-genome sequencing on an Illumina MiSeq platform. DNA dual-indexed libraries were generated using the Nextera XT 24 Index Kit (Illumina Inc., San Diego, CA, United States). After PCR amplification, the DNA amplicons were purified with the Agencourt AMPure XP beads (Beckman Coulter, Brea, CA, United States). The concentration of each amplicon was measured with the Qubit 2.0 Fluorometer (Life Technologies) and Qubit dsDNA HS Quantification kit, before normalization to create the pooled amplified library (PAL). Library quantification of the PAL was performed using the KAPA Library Quantification Kit (Kapa Biosystems Inc., Wilmington, MA, United States), and the concentrations were determined by qPCR. An appropriate dilution of the PAL was used according to the manufacturer’s recommended protocol for loading into an Illumina MiSeq v2 (2 × 150-bp paired-end reads) cartridge for sequencing.

### Genome Assembly and *in silico* Multi-Locus Sequence Typing

Raw FASTQ sequencing reads from the Illumina MiSeq whole-genome sequencing were processed using an assembly pipeline generated with the SeqSphere+ version 6 (Ridom GmbH, Münster, Germany)^4^. FastQC (Andrews et al., 2010) was used to perform a quality check of the read files to assess the sequencing quality scores, total number of reads, and GC content. For the removal of the Nextera XT index library adapters, Trimmomatic (Bolger et al., 2014) was applied to achieve an average Q score of 30 in a sweeping window of 20 bases. The BWA plug-in in the SeqSphere+ software was used for the assembly of genome sequences of each isolate by mapping the paired-end reads to the complete reference genome of *E. faecium* DO (TX16_NC-017960) (Qin et al., 2012). The resultant contiguous consensus sequences (contigs) were exported for *in silico* identification of vancomycin-resistance (*van*) locus using the ResFinder server on the Centre for Genomic Epidemiology (CGE) online tool^5^. The settings used were a minimum sequence identity threshold of 90% and a genome length identity cut-off of 60%. The assembled genome sequences were queried against the MLST tool^6^ from the CGE database to determine the sequence type of the isolates. The *E. faecium* MLST database^7^ was also queried to confirm the sequence types.

### Genome Analysis and Phylogenetic Comparison

A core genome MLST (cgMLST) scheme for *E. faecium* has been defined in the cgMLST database^8^ and imported into SeqSphere+. While conventional MLST is based on seven putative house-keeping genes, the cgMLST scheme utilizes 1,423 target genes thereby providing a higher level of discrimination between isolates (De Been et al., 2015). Distance calculations based on the number of allelic differences between isolates were used to detect clusters within given sequence types and this analysis was visualized using a minimum spanning tree in SeqSphere+.

Pairwise SNP analysis was then conducted to establish the phylogenetic relationship between cgMLST-clustered isolates of VREfm from the state’s public hospitals. Each isolate within a cluster was nominated as the reference genome against which the raw FASTQ sequences of the other isolates were assembled and a core SNP alignment was generated using Snippy^9^. The presence of a SNP was defined using a minimum nucleotide variant frequency of 95% and a minimum read depth of 20. Gubbins^10^ was used to process the resulting SNP alignment to predict regions of homologous recombination within each isolate cluster. This resulted in the generation of three separate SNP scores for the phylogenetic comparison of isolates: Total number of SNPS; Number of SNPs in homologous recombinant regions; and Number of SNPs in non-homologous recombinant regions. From the Gubbins output, a maximum-likelihood phylogenetic tree was also generated using PhyML with the generalised-time-reversible (GTR) model. The previously described recombination-filtered SNP threshold of ≤16 SNPs for VREfm was used as a guide for identifying clonally related or non-unique isolates (De Been et al., 2015; Schurch et al., 2018).

To confirm any epidemiological linkage between phylogenetically related isolates, the genomic data were integrated with clinical data including date of hospital admission, VREfm screening, date of hospital discharge, and patient movement records. Spatio-temporal analyses derived from this information were used to infer phylogenetic relationships and identify possible, probable, or unlikely instances of VREfm transmission.

## Results

### MLST Sequence Types and *van* Resistance Loci

A total of 257 VREfm isolates were collected from Tasmania’s two largest public hospitals, LGH (*n* = 176) and RHH (*n* = 81), during 2014 (*n* = 18), 2015 (*n* = 40) and 2016 (*n* = 199). The higher number of isolates collected at the above hospitals in 2016 compared to 2014 and 2015 reflects the previously reported increasing number of VREfm isolates collected in Tasmania through this period (Wilson et al., 2017). A higher proportion of isolates were from clinical specimens at LGH (*n* = 54, 30.7%) compared to the RHH (*n* = 14, 17.3%). *In silico* analyses of the VREfm isolates were performed to determine their multi-locus sequencing types (MLST) and confirm the vancomycin resistance (*van*) loci present.

**FIGURE 1:**
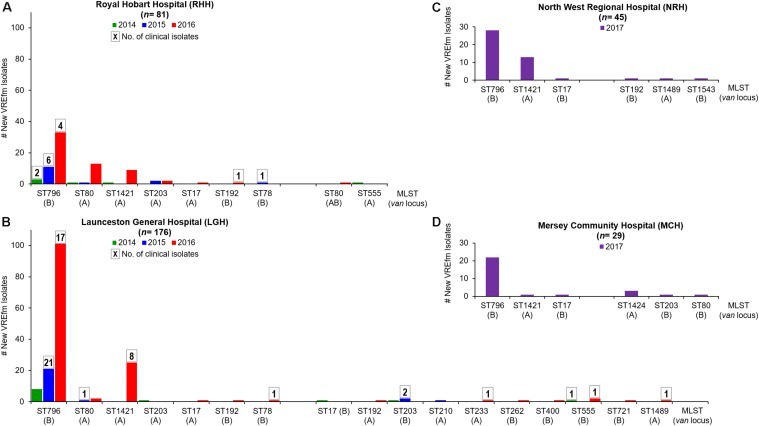
Multi-Locus Sequence Types (MLST) of vancomycin-resistant *Enterococcus faecium* (VREfm) isolates collected at Tasmania’s four acute public hospitals: **(A)** Royal Hobart Hospital (RHH) and **(B)** Launceston General Hospital (LGH) from 2014 to 2016; **(C)** North West Regional Hospital (NRH) and **(D)** Mersey Community Hospital (MCH). The MLST (and vancomycin-resistance locus) of the VREfm isolates were determined *in silico* from whole genome sequence data. Boxed numbers above histograms represent the number of new clinical VREfm isolates collected within each sequence type.

The two hospitals shared seven common sequence types, however, isolates from LGH exhibited a wider range of sequence types (*n* = 17 STs) compared to RHH (*n* = 9 STs) (Figure 1). The dominant *vanB*-harboring sequence type at both hospitals was ST796. For *vanA* VREfm isolates, more isolates at the RHH belonged to ST80 (*n* = 15) than the recently described ST1421 (*n* = 10) (Leong et al., 2018b). At LGH, most of the *vanA* isolates were ST1421 (*n* = 26) which accounted for 72.7% of *vanA* clinical isolates at that hospital. While all of the clinical cases from RHH harbored only the *vanB* locus, there was a mixture of *vanA* and *vanB* resistance loci among clinical isolates from LGH (Figure 1).

All of the NRH and MCH isolates (*n* = 74) analyzed were obtained from screening specimens and none were clinical isolates. Nine sequence types were represented among the isolates. The two most dominant sequence types were ST796-*vanB* (67.6%) and ST1421-*vanA* (24.3%) (Figure 1). ST1424-*vanA* was unique to MCH and was not isolated at NRH, LGH or RHH during the period analyzed.

### cgMLST Analysis of Tasmanian VREfm Isolates

**FIGURE 2:**
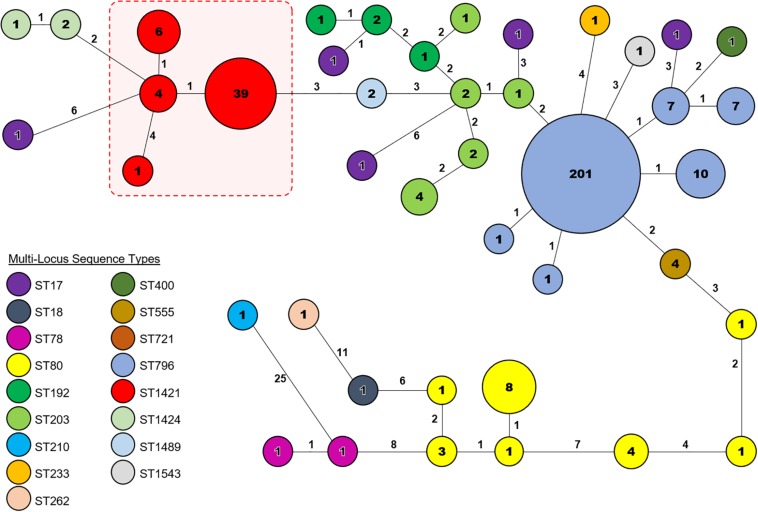
Core genome Multi-Locus Sequence Type (cgMLST) analysis of vancomycin-resistant *Enterococcus faecium* (VREfm) isolates from the four public hospitals in Tasmania (RHH, Royal Hobart Hospital; LGH, Launceston General Hospital; NRH, North West Regional Hospital; MCH, Mersey Community Hospital). The isolates were analyzed using cgMLST in Ridom SeqSphere+ and are visualized in a minimum spanning tree. Isolates with zero allele differences between them grouped together in clusters with isolates per cluster shown in the circles. The number of different alleles between clusters and unique isolates is shown on the connecting lines (not to scale).

**FIGURE 3:**
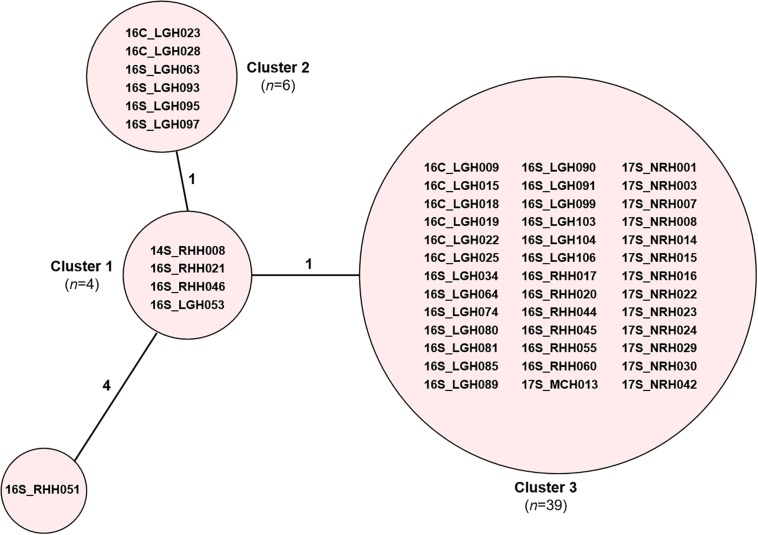
cgMLST of ST1421 vancomycin-resistant *Enterococcus faecium* (VREfm) isolates (*n* = 50). VREfm isolates belonging to sequence type ST1421 were analyzed by cgMLST in Ridom SeqSphere+ and are visualized in a minimum spanning tree. The isolates further differentiated into three clusters and one unique isolate (grouped into circles). The number of different alleles between clusters and the unique isolate is shown on the connecting lines (not to scale).

Core genome MLST resolved the dominant *vanB* sequence type in Tasmania, ST796, into one large cluster (*n* = 201) and three smaller clusters that differed by between 1 and 2 alleles, and two unique isolates (Figure 2). The dominant *vanA* sequence type at the RHH, ST80, separated into three cgMLST clusters that differed by up to 8 alleles, and four unique isolates. Application of cgMLST further differentiated the dominant *vanA* sequence type at LGH, ST1421, into three clusters which differ from one another by 1–2 alleles, and one unique isolate (Figure 2). The smallest cluster, Cluster 1, consists of three isolates from RHH and one isolate from LGH (Figure 3). Cluster 2 contains six isolates all of which were collected at LGH. The largest cluster, Cluster 3 (*n* = 39), includes isolates from all four of the state’s public hospitals (Figure 3).

### SNP Variant Analyses of Tasmanian VREfm Isolates

**FIGURE 4:**
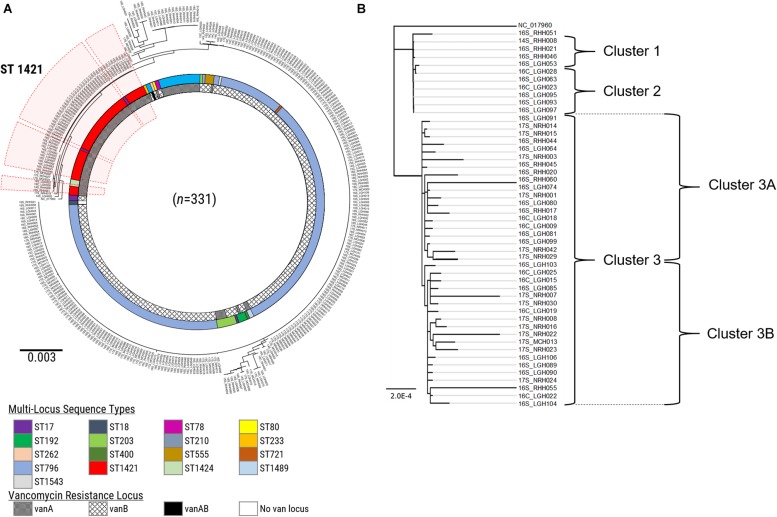
SNP-based analysis of vancomycin-resistant *Enterococcus faecium* (VREfm) isolates. **(A)** A SNP-based maximum-likelihood (PhyML) phylogenetic tree was generated from the VREfm isolates collected from the four public hospitals in Tasmania (RHH, Royal Hobart Hospital; LGH, Launceston General Hospital; NRH, North West Regional Hospital; MCH, Mersey Community Hospital) that were whole genome sequenced (*n* = 331) with reference genome *Enterococcus faecium* DO (TX16_NC-017960) to root the tree. Multi-locus sequence types (MLST) and vancomycin (*van*)-resistance loci are indicated. The clade of ST1421 isolates is highlighted in red. **(B)** Clades from the PhyML phylogenetic tree of VREfm isolates belonging to multi-locus sequence type ST1421 (*n* = 50) show concordance with the clusters determined by core-genome MLST (cgMLST) with Cluster 3 sub-dividing further into Clusters 3A and 3B.

A further refinement of the ST1421 VREfm isolates was performed by analyzing the differences on a nucleotide-base level to identify clonally related isolates. The genome of *E. faecium* DO (TX16_NC-017960) was used as the reference to generate a maximum-likelihood phylogenetic tree in PhyML based on recombination-filtered SNP differences between isolates (Figure 4). Firstly, there was good concordance between the SNP-based analysis and the cgMLST method with regard to the identification of clades of phylogenetically related isolates. For example, all of the ST1421 VREfm isolates positioned together based on the SNP differences. In addition, three main clusters of ST1421 isolates were evident from the phylogenetic tree (Figure 4). Closer examination of ST1421 revealed exact matches for isolate composition of Clusters 1 and 2 generated by both cgMLST and SNP analyses (Figure 4). Cluster 3 from cgMLST was further sub-divided into Clusters 3A and 3B by SNP analysis (Figure 4). The higher resolution of SNP variant analysis was then combined with epidemiological data on individual cases of VREfm from the four public hospitals in Tasmania. For comparison, SNP variant analyses were also conducted for the isolates belonging to sequence types ST80 (Supplementary Figures S3, S4) and ST796 (Supplementary Figures S5, S6). For the ST796 clusters, representative isolates (*n* = 31) were selected from the clades identified in the SNP-based phylogenetic tree (Supplementary Figure S5).

### Genomic and Epidemiological Analyses of ST1421 Cluster 1

**FIGURE 5:**
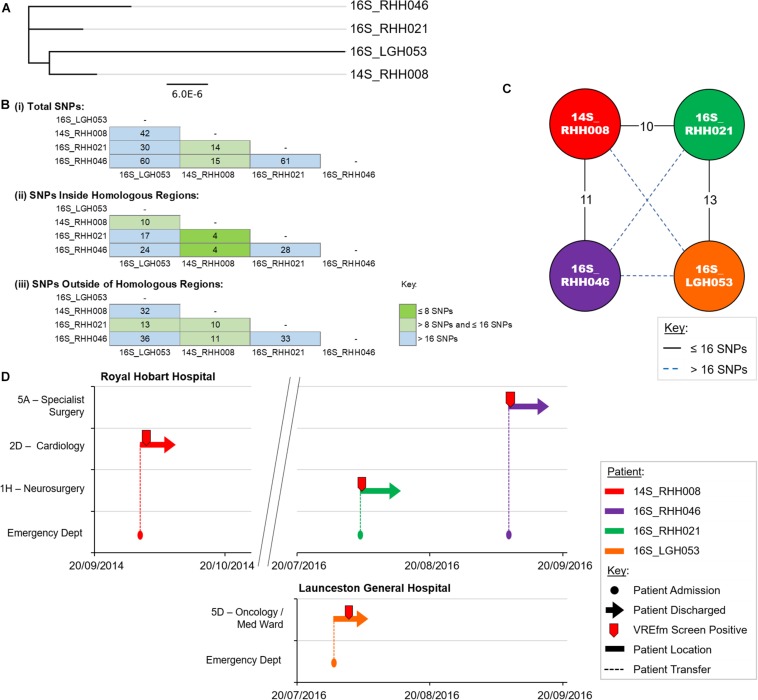
Phylogenetic analysis of vancomycin-resistant *Enterococcus faecium* (VREfm) isolates from ST1421 Cluster 1. **(A)** A SNP-based maximum-likelihood (PhyML) phylogenetic tree. **(B)** Matrix of pairwise comparison of SNP differences between isolates expressed as: (i) Total number of SNP differences; (ii) Number of SNPs inside homologous recombination regions, and (iii) Number of SNPs outside of homologous recombination regions. The previously described recombination-filtered SNP threshold of ≤16 SNPs for VREfm has been used as a guide for identifying clonally related or non-unique isolates. **(C)** Overview of recombination-filtered SNPs between isolates. The numbers of different SNPs between the isolates are shown on the solid black connecting lines. SNP differences above the threshold of 16 SNPs are shown as blue dotted lines. **(D)** Spatio-temporal location of patients in Cluster 1 who tested positive for VREfm at the Royal Hobart Hospital and Launceston General Hospital. The movement of patients following admission to the Royal Hobart Hospital through to date of discharge are indicated with respect to time (*x*-axis) and hospital ward location (*y*-axis). Each line color represents an individual patient. Patient 14S_RHH008 was admitted to RHH on September 30, 2014 and was screened for VREfm on October 1, 2014 from which a positive test was reported.

Cluster 1 contains four VREfm isolates with three collected at the RHH and one at LGH. The first patient in Cluster 1, 14S_RHH008 was transferred to RHH from an out-of-state hospital on September 30, 2014 and the VREfm isolate collected represents the first confirmed isolate of ST1421 in Tasmania (Figure 5). Approximately 2 years after patient 14S_RHH008 was discharged from RHH, other isolates also belonging to Cluster 1 were detected at the RHH and the LGH (Figure 5). At the RHH, isolates 16S_RHH021 and 16S_RHH046 were collected from VREfm colonized patients who were admitted to the Neurosurgery Ward and Specialist Surgery Ward, respectively. These isolates exhibited no cgMLST allele differences and ≤16 SNP differences with respect to each other and isolate 14S_RHH008. This indicates that further members of this cluster may exist but were not available in the isolate set, which may in part be reflective of VREfm sampling being more limited during 2014 and 2015 compared to 2016.

### Genomic and Epidemiological Analyses of ST1421 Cluster 2

**FIGURE 6:**
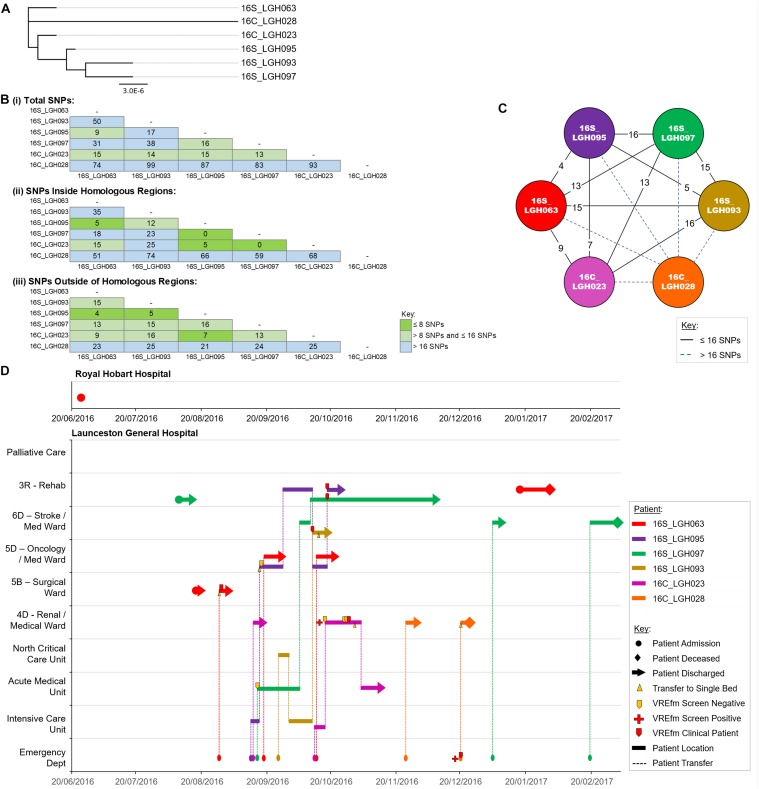
Phylogenetic analysis of vancomycin-resistant *Enterococcus faecium* (VREfm) isolates from ST1421 Cluster 2. **(A)** A SNP-based maximum-likelihood (PhyML) phylogenetic tree. **(B)** Matrix of pairwise comparison of SNP differences between isolates expressed as: (i) Total number of SNP differences; (ii) Number of SNPs inside homologous recombination regions, and (iii) Number of SNPs outside of homologous recombination regions. The previously described recombination-filtered SNP threshold of ≤16 SNPs for VREfm has been used as a guide for identifying clonally related or non-unique isolates. **(C)** Overview of recombination-filtered SNPs between isolates. The numbers of different SNPs between the isolates are shown on the solid black connecting lines. SNP differences above the threshold of 16 SNPs are shown as blue dotted lines. **(D)** Spatio-temporal location of patients in Cluster 2 who tested positive for VREfm at the Launceston General Hospital. The movement of patients following admission to hospital through to date of discharge are indicated with respect to time (*x*-axis) and hospital ward location (*y*-axis). Each line color represents an individual patient. As illustrated, patient 16S_LGH063 had admissions to both the Royal Hobart Hospital and Launceston General Hospital but was confirmed VREfm positive at the latter hospital.

Cluster 2 contains six isolates that were collected from patients when they were admitted to LGH. The first patient in the cluster, patient 16S_LGH063, was previously an out-patient at RHH on June 30, 2016 (Figure 6). On the patient’s second admission to the Surgical Ward at LGH, a VRE screening test on August 28, 2016 returned positive. Patient 16S_LGH063’s subsequent admissions were at the Medical Ward at LGH, and at both times, shared the same ward with a second patient, 16S_LGH095. Patient 16S_LGH095 initially tested negative for VREfm upon admission but subsequently tested positive after sharing the Medical Ward with patient 16S_LGH063 (Figure 6). 16S_LGH095 then shared the Rehabilitation Ward with a third patient, 16S_LGH097, who tested positive for VREfm after a previous negative result at earlier hospital admission. The overlaps in both time and ward location in the hospital for patients 16S_LGH063, 16S_LGH095, and 16S_LGH097, combined with no cgMLST allele differences and ≤16 SNP differences between their respective isolates, indicate that they belong to a clonally related outbreak of ST1421.

### Genomic and Epidemiological Analyses of ST1421 Cluster 3A

**FIGURE 7:**
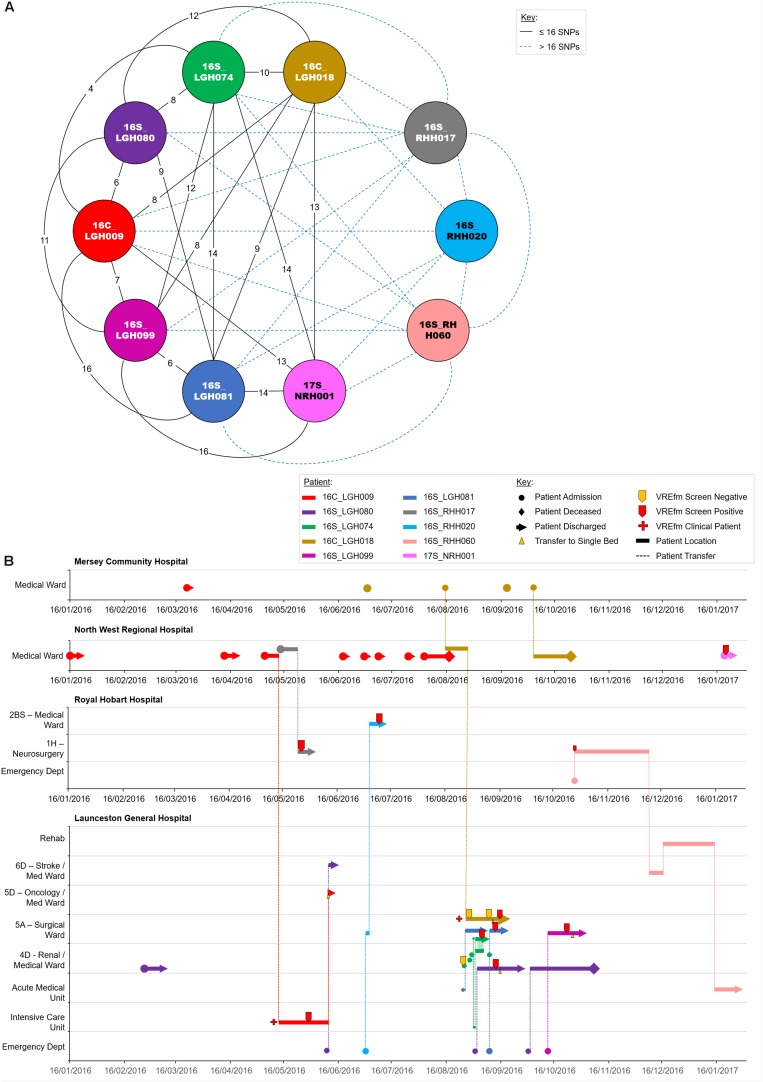
Phylogenetic analysis of vancomycin-resistant *Enterococcus faecium* (VREfm) isolates from ST1421 Cluster 3A. The previously described recombination-filtered SNP threshold of ≤16 SNPs for VREfm has been used as a guide for identifying clonally related or non-unique isolates. **(A)** Overview of recombination-filtered SNPs between isolates. The numbers of different SNPs between the isolates are shown on the solid black connecting lines. SNP differences above the threshold of 16 SNPs are shown as blue dotted lines. **(B)** Spatio-temporal location of patients in Cluster 3A who tested positive for VREfm. The movement of patients following admission to hospital through to date of discharge are indicated with respect to time (*x*-axis) and hospital ward location (*y*-axis). Each line color represents an individual patient. As illustrated, a number of patients had multiple admissions to more than one hospital over the time course.

The isolates in Cluster 3A were collected from patients who were admitted to LGH (*n* = 6), NRH (*n* = 4), and RHH (*n* = 3). There were a number of patients belonging to this cluster who had admissions to multiple hospitals, e.g., 16C_LGH009, 16S_LGH080, 16C_LGH018, 16S_RHH020, and 16S_RHH060 (Figure 7). The first patient, 16C_LGH009, was not screened for VREfm during the initial three admissions at NRH and MCH (Figure 7). The patient’s admission on May 5, 2016 at NRH involved an inter-hospital transfer to the Intensive Care Unit at LGH where a clinical sample collected on May 28, 2016 was confirmed positive for VREfm. During an admission to MCH on August 15, 2016, patient 16C_LGH018 was involved in two inter-hospital transfers to NRH (August 15, 2016) and LGH (August 27, 2016) (Figure 7). While staying in the Surgical Ward at LGH, the patient underwent three VREfm screenings. The initial two tests were negative, however, the third clinical isolate from patient 16C_LGH018 tested positive for VREfm. Spatio-temporal analysis indicated that two other patients, 16S_LGH074 and 16S_LGH081, who shared the Surgical Ward with patient 16C_LGH018, were also tested positive for VREfm on September 5, 2016 and September 12, 2016, respectively. Pairwise SNP-based analysis revealed that the VREfm isolates from these patients differed by ≤16 SNPs (Figure 7). Additionally, patient 16C_LGH018 was re-admitted at MCH and also at NRH, providing opportunities for further dissemination of VREfm beyond the initial hospital where the infection was first confirmed (Figure 7). Pairwise SNP differences between the isolates in Cluster 3A are shown in Supplementary Figure S1.

### Genomic and Epidemiological Analyses of ST1421 Cluster 3B

**FIGURE 8:**
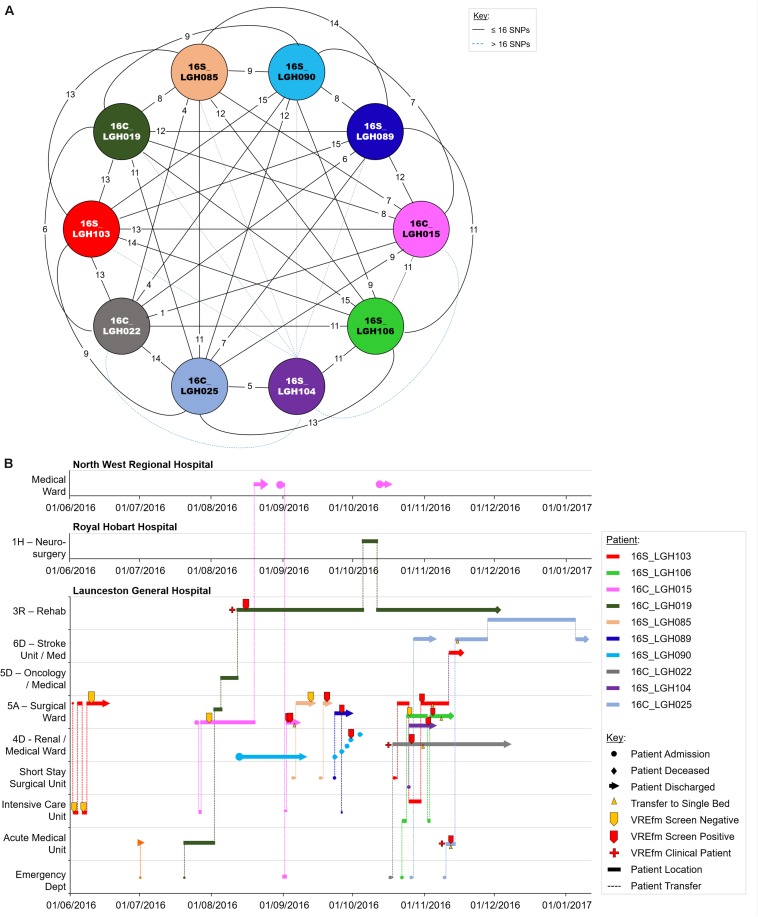
Phylogenetic analysis of vancomycin-resistant *Enterococcus faecium* (VREfm) isolates from ST1421 Cluster 3B. The previously described recombination-filtered SNP threshold of ≤16 SNPs for VREfm has been used as a guide for identifying clonally related or non-unique isolates. **(A)** Overview of recombination-filtered SNPs between isolates. The numbers of different SNPs between the isolates are shown on the solid black connecting lines. SNP differences above the threshold of 16 SNPs are shown as blue dotted lines. **(B)** Spatio-temporal location of patients in Cluster 3B who tested positive for VREfm. The movement of patients following admission to hospital through to date of discharge are indicated with respect to time (*x*-axis) and hospital ward location (*y*-axis). Each line color represents an individual patient. As illustrated, a number of patients had multiple admissions to more than one hospital over the time course.

Of the 10 isolates in Cluster 3B which were collected in LGH, seven patients tested positive for VREfm after staying in the Surgical Ward (Figure 8). The first patient, 16S_LGH103, was admitted to the Surgical Ward on June 1, 2016 and subsequently to the Intensive Care Unit where the patient tested negative for VREfm. However, during a second admission to the Surgical Ward at LGH, the patient tested positive for VREfm on October 30, 2016. Spatio-temporal analysis indicated that two other patients, 16S_LGH106 and 16S_LGH104, shared the Surgical Ward with patient 16S_LGH103 and tested positive for VREfm on November 1, 2016 and November 3, 2016, respectively (Figure 8). Pairwise SNP-based analysis revealed that isolates from patients 16S_LGH103 and 16S_LGH106 differed by ≤16 SNPs and zero cgMLST alleles indicating that they are clonally related (Figure 8).

As in the case of patient 16S_RHH060 in Cluster 3A, patient 16C_LGH019 from Cluster 3B, underwent an inter-hospital transfer after testing positive for VREfm, providing a further example of the propensity for inter-institutional spread ofthe ST1421 sequence type in Tasmania. Pairwise SNP differences across Cluster 3B are shown in Supplementary Figure S2.

## Discussion

In this study, we established the sequence types of VREfm isolated at the four public hospitals in Tasmania. While both the RHH and LGH shared ST796 as their dominant *vanB* sequence type, interestingly, the two hospitals exhibited a different profile with respect to other sequence types present among isolates collected from 2014 to 2016 (Figure 1). For example, while ST80 was the prominent *vanA* VREfm at the RHH, a limited number of ST80 isolates were collected at the LGH where instead, the recently discovered ST1421 was more dominant. All of the clinical isolates at the RHH belonged to *vanB* sequence types, whereas both *vanA* and *vanB* sequence types constituted clinical isolates obtained at the LGH. The identification of ST1421 in Tasmania appears to coincide with the change in Australia from a near-complete dominance by the *vanB* resistance locus among VREfm to an expansion of isolates that harbor the *vanA* resistance locus from only 1.9% (2/107) of vancomycin non-susceptible *E. faecium* bloodstream isolates in 2011 to 43.0% (83/193) by 2016 (Coombs et al., 2014a, Coombs et al., 2018). In this Tasmania-wide study, the collection of 331 clinical and overlapping-screening isolates consisted of 74.6% *vanB* (*n* = 247), 25.1% *vanA* (*n* = 83), and 0.3% *vanAB* (*n* = 1).

**FIGURE 9:**
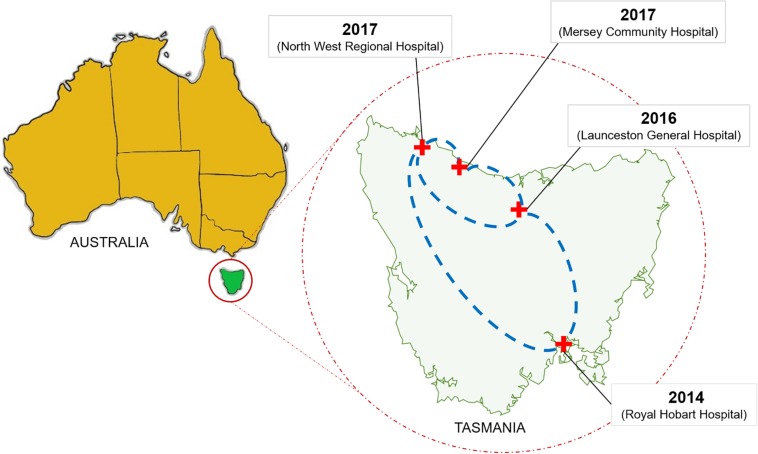
Dissemination of the *vanA* ST1421 vancomycin-resistant *Enterococcus faecium* (VREfm) in Tasmania’s public hospital system. The first reported case of ST1421 VREfm in Tasmania occurred at the Royal Hobart Hospital in 2014. Correlation of genomic data with epidemiological data revealed a network in which recently identified sequence type of VREfm (ST1421) emerged in each of the state’s other public hospitals.

Our whole-genome sequence data were then applied to cgMLST and SNP analyses of the 331 VREfm isolates. This revealed the existence of three cgMLST clusters within ST1421 which resolved further into clusters (1, 2, 3A, and 3B) based on SNP-variant analyses (Figure 4). When we combined the genomic data with patient spatio-temporal information, a number of features of VREfm epidemiology in Tasmania became evident. Firstly, with regard to Clusters 2, 3A, and 3B, clonally related isolates which differed by ≤16 SNPs and zero cgMLST alleles were collected from patients who shared specific hospital wards at the same time, indicating potential intra-institutional transmission involving these patients (Figures 6–8). Secondly, with respect to Clusters 3A and 3B, patients who were confirmed positive for VREfm infection or colonization at one hospital, were subsequently transferred or re-admitted to another hospital in Tasmania which provided opportunities for onward inter-institutional spread of VREfm in the state (Figures 7, 8). Lastly, Cluster 1 contains the first confirmed isolate of ST1421 in Tasmania. The patient was transferred from an out-of-state hospital to a Tasmanian hospital in 2014 shortly before testing positive for VREfm at the latter hospital. This indicates potential inter-state transmission of a newer VREfm sequence type. Subsequently, ST1421 started to be isolated at the other public hospitals in Tasmania from 2016 onward (Figure 9) and it is highly possible that this state-wide spread involved movements of VREfm-positive patients between locations when taking into account the collection of clonally related isolates across multiple hospitals.

Risk factors associated with VRE colonization have been found to include exposure to any antibiotic, diarrhea, and longer length of stay in hospital (Karki et al., 2012). Furthermore, intensive care admission, a higher burden of co-morbidities, and longer time to appropriate antibiotics have been associated with mortality in enterococcal bacteremia (Cheah et al., 2013). A study of 103 patients with confirmed VREfm infection or colonization found that 40% of patients remained positive in the first year of follow-up and that 23.3% were still positive in the fourth year of follow-up (Karki et al., 2013). While the investigators observed a downward trend in fecal carriage of VREfm over time, the findings revealed that, even in the absence of recent risk factors including hospitalization or antibiotic use, patients with a previous history of VREfm can harbor the pathogen for a period in the order of years. This implies that patients discharged from one hospital may still harbor VREfm, and therefore be potentially infectious, when admitted to another healthcare institution in another jurisdiction several months or even years later. The repatriation of a VREfm-positive patient has been linked to the regional spread of a sequence type, ST796, from a hospital in Melbourne, Australia to a hospital in Auckland, New Zealand (Mahony et al., 2018). In addition, a recent outbreak of VREfm in hospitals in Switzerland highlights the potential for new sequence types to move globally. ST796 had not been reported in Switzerland prior to 2017. However, between December 2017 and April 2018, four hospitals in the Canton of Bern isolated this sequence type from 89 patients. Markedly, 77 out of the 89 isolates (86.5%) belonged to ST796 with the remaining isolates made up of ST117 (*n* = 6), ST78 (*n* = 4), ST555 (*n* = 2), ST17 (*n* = 1), and ST80 (*n* = 1) (Wassilew et al., 2018). The findings suggest a relatively recent introduction of ST796 into Switzerland and its subsequent establishment as a dominant sequence type.

The origins of ST796 can be traced back to Australia, where it was first discovered in 2012, and subsequently identified as the source of a notable increase in VREfm colonization at a Melbourne neonatal intensive care unit in 2013 (Lister et al., 2015). By 2015, ST796 had become the dominant *vanB* sequence type among patient episodes of *E. faecium* bacteremia in Melbourne hospitals (Buultjens et al., 2017) displacing the previously endemic *vanB* sequence type ST203 (Coombs et al., 2014b). The ability of ST796 to establish relatively quickly in new geographical locations and out-compete existing strains of VREfm suggests the potential existence of inherent advantageous properties in this sequence type. Indeed, generation of a complete genome sequence for an ST796 isolate revealed that it likely evolved from an ST555-like ancestral progenitor through the acquisition of transposons Tn*1549* and Tn*916* conferring resistance to vancomycin and tetracycline, respectively, along with plasmids, prophages, cryptic genome islands, and chromosomal SNPs (Buultjens et al., 2017). Similarly, the recently described ST1421 VREfm strain has been identified as a variant of the ST17 strain due to a mutation in the housekeeping gene, *pstS*, that is used for MLST (Andersson et al., 2019). Previous studies attributed this occurrence to multiple recombination events in Australia (Van Hal et al., 2018) and also the insertion of a Tn*5801*-like transposon into the *tetM* gene, an event commonly detected in *vanA* VREfm strains that have lost the *pstS* locus (Lemonidis et al., 2019). In addition to antibiotic resistance, it is believed that the new gene content has conferred adaptations to the healthcare environment on ST796. One such adaptation may include higher tolerance to isopropanol used in hospital hand hygiene products that was reported in a number of recently emerged sequence types of *E. faecium* (Pidot et al., 2018).

A previous study has shown that hand-hygiene measures are only effective when used in combination with other interventions to control the transmission of VREfm (Wolkewitz et al., 2008). Environmental contamination remains an important factor in spread due to the ability for VREfm to persist on surfaces for prolonged periods of time (Wendt et al., 1998). A recent multi-center randomized trial, REACH, involving 1,700 environmental services staff and 6,100 overnight beds across 11 hospitals in Australia found that interventions with regard to improved cleaning techniques, disinfectant products used, staff training, auditing, and communication for routine hospital cleaning increased the percentage of cleaned frequent touch points from 55% to 76% in bathrooms and from 64% to 86% in bedrooms (Mitchell et al., 2019). Although colonizations were not assessed, the interventions were associated with a reduction in clinical VRE infections from 0.35 to 0.22 per 10,000 occupied bed days (Mitchell et al., 2019). An earlier study performed at one hospital in Melbourne found that the use of a bleach-based cleaning-disinfection program correlated with a decrease in both VRE colonizations in high-risk patients and VRE bacteremia cases (Grabsch et al., 2012).

In summary, based on available evidence, it is apparent that the marked increase of VREfm in Tasmania involved factors that included the emergence of newer sequence types in the state and also the movement of infected or colonized patients between hospitals. This has important implications for VREfm control in Australia and further afield. Newly detected sequence types need to be carefully monitored and where necessary, targeted with enhanced strategies that include managing patients with transmission-based precautions. The Australian Guidelines for the Prevention and Control of Infection in Healthcare recommend measures such as placement of patient alerts and screening of patients with VREfm transferred within and between healthcare institutions (NHMRC, 2019). Coordination of efforts and knowledge between institutions is required when changes in genotypic profiles of dominant strains occur and when new sequence types emerge. For this, rapid routine identification of VREfm types, beyond standard vancomycin-resistance locus determination, is required. To be effective, this work will necessitate the use of whole-genome sequencing on a routine and real-time basis. Therefore, sequencing and bioinformatic protocols for VREfm will need to be standardized between laboratories to translate the technology from retrospective to real-time applications.

## Data Availability Statement

The datasets generated for this study can be found in the NCBI Sequence Read Archive repository, under the BioProject number PRJNA592871.

## Ethics Statement

Ethics approval for this study was obtained from the Tasmania Health and Medical Human Research Ethics Committee (Reference# H0016214).

## Author Contributions

RO conceived the study, obtained funding, supervised the work, analyzed the data, and drafted the manuscript. KL performed the laboratory experimentation and genome sequence analysis, and drafted the manuscript. RK and EL retrieved isolates and matched epidemiological information. LC, TA, and AW assisted in planning the study and manuscript review.

## Conflict of Interest

The authors declare that the research was conducted in the absence of any commercial or financial relationships that could be construed as a potential conflict of interest.

## Footnotes

1 http://www.eucast.org/clinical_breakpoints/

2 http://cdstest.net

3 http://www.cepheid.com/

4 http://www.ridom.de/seqsphere/

5 https://cge.cbs.dtu.dk/services/ResFinder/

6 http://cge.cbs.dtu.dk/services/MLST/

7 http://pubmlst.org/efaeciuim/

8 https://www.cgmlst.org/ncs/schema/991893/

9 https://github.com/tseemann/snippy

10 https://github.com/sanger-pathogens/Gubbins
