# Supplementary material for: State-Wide Genomic and Epidemiological Analyses of Vancomycin-Resistant Enterococcus faecium in Tasmania’s Public Hospitals
Source: Front Microbiol. 2020 Jan 15;10:2940. doi: 10.3389/fmicb.2019.02940 (PMC6975128; doi:10.3389/fmicb.2019.02940)
Supplement: Supplementary file 1 [file Data_Sheet_1.docx]

Supplementary Figure S1 | Pairwise comparisons between vancomycin-resistant *Enterococcus faecium* (VREfm) isolates from Cluster 3A, which measures the differences in unique SNPs on three differential scores: (i) Total number of SNP differences; (ii) Number of SNPs determined inside homologous recombination regions, and (iii) Number of SNPs determined outside of homologous recombination regions.

**Supplementary Figure S2** | Pairwise comparisons between vancomycin-resistant *Enterococcus faecium* (VREfm) isolates from Cluster 3B, which measures the differences in unique SNPs on three differential scores: (i) Total number of SNP differences; (ii) Number of SNPs determined inside homologous recombination regions, and (iii) Number of SNPs determined outside of homologous recombination regions.

Supplementary Figure S3 | SNP-based phylogenetic analysis of vancomycin-resistant *Enterococcus faecium* (VREfm) isolates belonging to multi-locus sequence type ST80 (*n*=19). A maximum-likelihood (PhyML) phylogenetic tree was generated with *Enterococcus faecium* DO (TX16_NC-017960) as the reference genome to root the tree.


Supplementary Figure S4 | Pairwise comparisons between vancomycin-resistant *Enterococcus faecium* (VREfm) isolates from Cluster 3B, which measures the differences in unique SNPs on three differential scores: (i) Total number of SNP differences; (ii) Number of SNPs determined inside homologous recombination regions, and (iii) Number of SNPs determined outside of homologous recombination regions.

Supplementary Figure S5 | SNP-based phylogenetic analysis of vancomycin-resistant *Enterococcus faecium* (VREfm) isolates belonging to multi-locus sequence type ST796 (*n*=227). A maximum-likelihood (PhyML) phylogenetic tree was generated with *Enterococcus faecium* DO (TX16_NC-017960) as the reference genome to root the tree. For SNP-pairwise comparison, representative VREfm ST796 isolates (highlighted in red boxes) from each clade in the phylogenetic tree were selected.

Supplementary Figure S6 | Pairwise comparisons between vancomycin-resistant *Enterococcus faecium* (VREfm) isolates belonging to ST796, which measures the differences in unique SNPs on three differential scores: (i) Total number of SNP differences; (ii) Number of SNPs determined inside homologous recombination regions, and (iii) Number of SNPs determined outside of homologous recombination regions.
